# A virtual reality intervention for fear of movement for Veterans with chronic pain: protocol for a feasibility study

**DOI:** 10.1186/s40814-019-0501-y

**Published:** 2019-12-11

**Authors:** Christopher A. Fowler, Lisa M. Ballistrea, Kerry E. Mazzone, Aaron M. Martin, Howard Kaplan, Kevin E. Kip, Jennifer L. Murphy, Sandra L. Winkler

**Affiliations:** 1 Research and Development Service, James A. Haley Veterans Hospital and Clinics, 8900 Grand Oak Circle, Tampa, FL 33705 USA; 2 James A. Haley Veterans Hospital and Clinics, 13000 Bruce B. Downs Blvd, Tampa, FL 33612 USA; 3 Advanced Visualization Center, University of South Florida – Information Technology, 4202 E. Fowler Avenue, CMC147, Tampa, FL 33620 USA; 4 College of Public Health, University of South Florida, 13201 Bruce B. Downs Blvd, MDC56, Tampa, FL 33612 USA; 5 Department of Neurology, University of South Florida, 12901 Bruce B. Downs Blvd, Tampa, FL 33612 USA

**Keywords:** Chronic pain, Virtual reality, Veterans, Rehabilitation, Fear of movement, Kinesiophobia, Exposure therapy, Distraction therapy, Oculus rift, Feasibility

## Abstract

**Background:**

A key concern for people with chronic pain is experiencing increased pain and/or re-injury. Consequently, individuals with chronic pain can develop a maladaptive fear of movement that leads to adverse functional consequences. A primary goal of chronic pain rehabilitation is re-engagement in feared movements through exposure. This is often challenging since safe movement can be uncomfortable. Virtual environments provide a promising opportunity to safely and gradually expose Veterans to movements that are avoided in the real world. The current study will utilize multiple virtual reality (VR) applications (APPs) of varying the intensity levels ranging from passive distraction from pain to active exposure to feared movement. The primary aims of this pilot are to examine VR as an adjunctive nonpharmacological intervention to assist with the adoption and implementation of skills to decrease fear of movement and increase overall functioning among Veterans with chronic pain. Second, to build a hierarchy of VR APPs to assist in gradual exposure to feared movements.

**Methods:**

This study will be conducted in the Chronic Pain Rehabilitation Program (CPRP) at the James A. Haley Veterans Hospital, a unique inpatient program within the VA system. Participants will include up to 20 Veterans who receive a VR intervention as part of their physical therapy. A rating form containing qualitative and quantitative experiences will be administered following each VR session to assess feasibility and to provide descriptive information for the proposed hierarchy. Effect sizes will be calculated from intake and discharge measures for the primary outcome fear of movement and secondary pain and functional outcomes.

**Discussion:**

This study will inform the feasibility of a randomized controlled trial examining the clinical utility of using VR to reduce fear of movement and increase function among Veterans with chronic pain. VR has the advantage of being easily implemented both within VA healthcare settings as well as in Veterans’ own residences, where engagement in ongoing self-management approaches is often most challenging. Presumably, VR that is matched to patient needs, progresses in intensity, immerses Veterans in the applications, and is perceived positively by Veterans, will result in positive functional outcomes.

## Background

Pain is among the most costly disorders treated in Department of Veterans Affairs (VA) settings [1]. Over five million Veterans were diagnosed with at least one musculoskeletal disorder from 2001 to 2011, nearly half of whom reported moderate-to-severe pain [2]. The treatment of pain was established as a VA priority in 1998 [3], but in recent years, pain management has received more attention due to national concerns regarding opioid overdose, addiction, and high-profile adverse events [4]. Given this burden, the Centers for Disease Control and Prevention [4] and other national organizations [5] have recommended non-pharmacological approaches as the preferred treatments for chronic non-cancer pain.

### Fear of movement

A key concern for people with chronic pain, defined as pain persisting longer than 3 months [6], is experiencing increased pain or (re) injury [7]. As a result, individuals often develop kinesiophobia, a fear of movement [7]. Kinesiophobia can be helpful in acute pain which lasts less than 3 months [6], but maladaptive in chronic pain, as it leads to adverse functional consequences [7]. A primary goal of chronic pain rehabilitation is re-engagement in feared movements through exposure. However, this is often challenging since safe movement can be physically and emotionally uncomfortable [8].

### Virtual reality

Virtual reality (VR) can be defined as “computer-generated simulations of three-dimensional objects or environments with seemingly real, direct, or physical user interaction” (pg. 34) [9]. Today’s virtual technologies use the computer and wearable devices to give the user the illusion of being immersed or present in a non-physical world [10, 11]. VR can serve as an adjunctive method in evidence-based interventions to assist with the delivery and adoption of self-management skills [11–13]. When VR that is matched to patient level of functioning progresses in intensity, immerses people in the applications, and is perceived positively, it can contribute to improved fear of movement and disability [12, 13]. To date, VR research [11–13] has prominently focused on two cognitive behavioral therapy [8] techniques, distraction from and exposure to pain.

### Virtual reality and pain distraction

Evidence supports the use of VR to attenuate pain with the majority of evidence to date aimed at treating acute pain. A rapid evidence assessment of immersive VR for acute pain management (17 studies, 337 patients) found strong evidence for immediate and short-term pain reduction, as well as moderate evidence for short-term analgesic effects on physical function [11]. Distraction-focused treatments (e.g., guided imagery, relaxation training) are the most commonly researched VR-administered interventions for acute pain relief [11, 14]. Distraction therapy is based on the assumption that humans have finite attentional resources [8, 15]. VR distraction is hypothesized to consume attention leaving less cognitive capacity for processing pain [15] and fear of movement [13]. A recent controlled trial conducted in an inpatient acute pain care setting demonstrated reduced pain scores among patients playing a “medium-intensity” pain distraction VR application (APP) compared to a televised high-definition nature video [16]. Pilot work suggests that relaxation using VR may be associated with reduced pain intensity among people with chronic pain [17].

### Virtual reality and exposure therapy for pain

In contrast to pain distraction, exposure therapy *focuses attention* on the fearful stimulus and inducing a feeling of “being there” [18, 19]. To be effective, exposure therapies should be graded, motivating, and related to real-life functional activities [14, 20]. A randomized study found that integrating exposure via guided virtual walking into physical therapy resulted in a significant decrease in fear of movement and pain intensity when compared to physical therapy without VR [21]. A feasibility study found high acceptability ratings of a virtual dodgeball intervention to influence lumbar flexion for people with chronic low back pain and pain-related fear [22]. No participants withdrew from the study and no adverse events or increased medication use was reported [22]. This evidence supports using VR as an adjunctive therapeutic delivery method for reducing fear of movement among people with chronic pain [21, 22].

### Hierarchy from distraction to exposure

It has been argued that distraction from pain-related thoughts and emotions can promote avoidance of factors contributing to the development and maintenance of chronic pain [12, 13]. This can undermine the effectiveness of distraction-only VR therapies among chronic pain populations who have a greater need for rehabilitation than immediate relief [12, 23]. We have conceptualized a two-dimensional hierarchy for movement and intensity of stimulation for people with chronic pain. The hierarchy will range from low-intensity pain distraction to more “interactive” graded exposure techniques within the same VR-assisted intervention (e.g., sitting passively in a chair during guided meditation to standing and using body movement to engage in virtual activities such as painting).

### Current study

The proposed project is informed by the Fear-Avoidance Model of Chronic Pain [7] and assumes that as people gradually confront feared activities through VR, maladaptive pain beliefs are challenged and fear responses are extinguished [14, 24]. Both distraction [22] and exposure [21] could be beneficial for improving fear of movement among people with chronic pain. Still, studies typically examine these methods independently despite the former being inherent during VR utilization [14]. This will be the first known study that will investigate the use of immersive VR across a distraction to exposure spectrum as an adjunct strategy for chronic pain management in a sample of US Veterans.

## Aims and objective

The objective of this study is to establish feasibility for a future randomized controlled trial (RCT) that will test the effectiveness of VR as a treatment adjunct for chronic pain management and aim to validate the proposed hierarchy. The primary aims are as follows:

Aim 1: Describe the individual Veteran trajectories and APP intensity ratings on the proposed distraction-to-exposure hierarchy.

- RQ 1.1: How many different trajectory patterns emerge as Veterans progress across the intensity levels of the distraction-to-exposure hierarchy?
- RQ 1.2: How do Veterans rate the intensity of the VR APPs?

Aim 2: Estimate the within-subjects effect size and 95% confidence interval (CI) associated with changes in fear of movement and secondary outcomes to provide insight into the likely magnitude of effect associated with the VR intervention.

- RQ 2.1: What is the estimated within-subjects effect size and 95% CI for changes in the primary outcome, fear of movement, from baseline to post-test following the VR intervention?
- RQ 2.2: What is the estimated within-subjects effect size and 95% CI in *common* feared movements from baseline to post-test following the VR intervention?
- RQ 2.3: What are the within-subjects effect sizes and 95% CIs for changes for the secondary outcomes of pain intensity, pain interference, pain catastrophizing, pain-related functioning, and negative affect from baseline to post-test following the VR intervention?
- RQ 2.4: What proportion of Veterans experience clinically meaningful change for the following outcomes: common feared movements, pain intensity, pain catastrophizing, pain-related functioning, and negative affect?

Aim 3: Pilot test this protocol to assess the feasibility of VR use to plan for a future randomized controlled trial.

- RQ 3.1: How do Veteran users describe their experiences with VR during this study?
- RQ 3.2: What are the identified barriers and facilitators of VR use?
- RQ 3.3: What is the estimated compliance (percentage of sessions attempted, completed) with VR?

## Methods

### Study setting

The CPRP at the James A. Haley Veterans Hospital is a 19-day residential chronic pain treatment program. The interdisciplinary treatment provided in the CPRP utilizes a cognitive behavioral treatment approach that targets the physical and psychological impact of chronic pain. The CPRP is the sole inpatient chronic pain program within the VA system, and its attendees are Veterans referred system-wide.

### Participants and recruitment

All Veterans (*N* ≤ 20) enrolled in the CPRP over the 3-week data collection period will be recruited. Interested individuals will be consented in-person by the research staff. Given the pragmatic nature of this study, eligibility is consistent with the CPRP program. Inclusion criteria: (1) the presence of chronic pain syndrome (ICD-9-CM code 338.4) and experiencing psychosocial dysfunction and functional impairments due to chronic pain and (2) a negative urine drug screen for alcohol, illicit substances, and prescribed opioid or psychiatric medications. Exclusion criteria: (1) uncontrolled depressive symptoms; (2) actively suicidal; (3) uncontrolled psychotic symptoms; (4) a recent history of violent or aggressive behavior; (5) high fall risk; and (6) cardiac, pulmonary, or neurological contraindications [25, 26].

Each week, four Veterans graduate from the program and are discharged from the CPRP. Up to four new Veterans are then admitted into the CPRP. Enrollment includes up to 12 Veterans at a time. Demographic characteristics from a CPRP cohort study (*N* = 705) reported that Veterans were 50.08 ± 11.03 years old with an average pain chronicity of 13.02 ± 10.85 years, report baseline pain near the “severe” range (7/10) on a numeric rating scale 6.95 ± 1.65, primarily experience back pain (56.20%), are prominently male (79.80%), Caucasian (60.77%), on disability or retired (73.22%), slightly more likely to be married (51.63%), less likely to be prescribed opioids daily (39.46%), and experience “moderate” relief from opioid therapy [25]. The program attrition rate for non-compliance during this period was 14.89% (*N* = 105). Consistent with the VA mission to reduce high-dose opioid use, all Veterans must agree to begin an opioid taper upon CPRP admission when applicable.

### Design

A type 1 hybrid design will be used to blend clinical effectiveness and implementation research to accelerate the proposed VR intervention into practice. Primary and secondary outcomes (Aim 2) will be assessed using a within-subjects pretest-posttest design. Qualitative pre-implementation data will be collected following each VR session. The VR intervention will be implemented as an adjunct part of graded exposure physical therapy (PT) sessions in the CPRP. For each session, participants will be assigned to use one of two VR head-mounted displays (HMD: Oculus Rift, Gear VR). VR assignment will be balanced secondary to equipment availability. Specifically, participants will participate in two sessions of Gear VR [27] for each session of Oculus Rift [28].

### Intervention

To build a hierarchy of VR APPs for matching the technology to patient need, the VR intervention will consist of 12 commercially-available APPs (six per VR modality) selected by investigators that could potentially reduce fear of movement. The APPs are matched based on two dimensions: movement intensity (low, moderate, high) and Veteran position (seated, standing). Movement intensity includes the level of movement required to meet the demands of the APP. Low-intensity APPs will require minimal movement activities including guided meditation and visual imagery-based environments. Moderate intensity APPs include more active demands including exploring virtual environments and controlling air or watercraft. High-intensity APPs will require participants to use a greater range of bodily motion including painting on 3D canvases or rhythm-based activities (similar to the video game “Rock Band”) [29]. While previous studies have emphasized the intensity [16], it is important to consider whether the participant uses the VR technology in a seated vs. a standing position because this may impact the intensity of the required movement. The APPs utilized in this study [30–40] are presented in Table 1.

**Table 1:** APPs for Oculus Rift VR and Samsung Oculus Gear VR by intensity

Intensity	VR HMD	
	Oculus Rift	Samsung Oculus Gear VR
Low	(1) Guided Meditation VR [30] (2) Perfect [31]	(1) Guided Meditation VR [30] (2) Rest VR: Rest & Meditate [36]
Medium	(3) Nature Treks VR [32] (4) The Grand Canyon VR Experienc e[33]	(3) Ocean Rift [37] (4) Reveries: Dream Flight [38]
High	(5) Tilt Brush [34] (6) The Show Must Go On [35]	(5) Paint VR [39] (6) Beats Fever Paper [40]

**Fig. 1:**
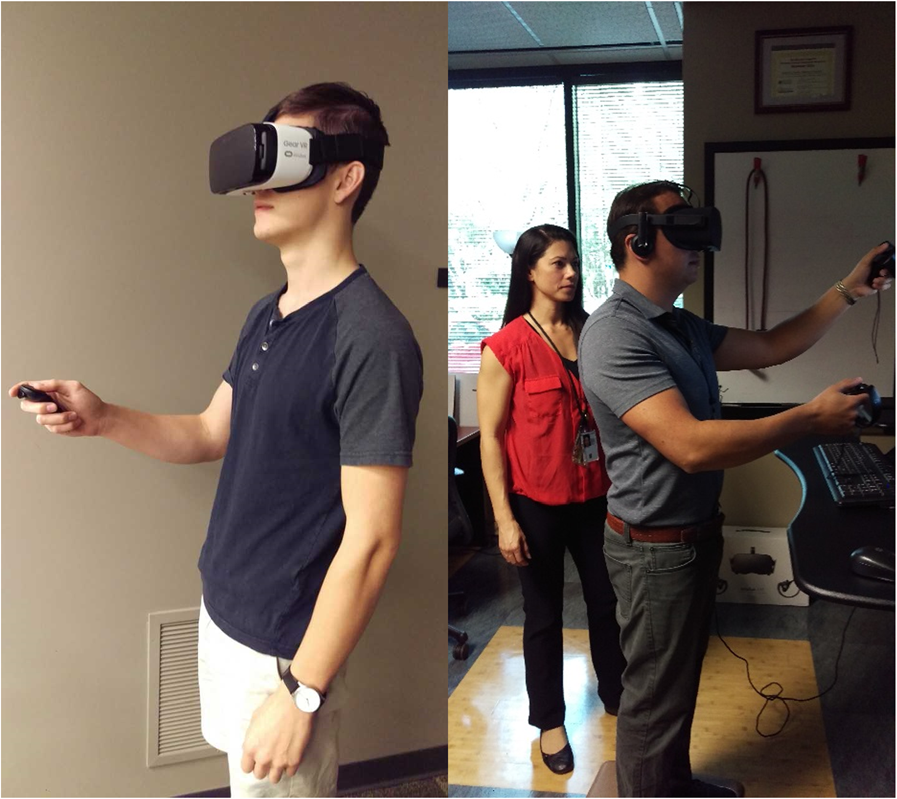
Samsung Oculus Gear VR with supplemental hand controller (left) and Oculus Rift (right)

Comparable APPs were chosen for two VR HMDs, Oculus Rift VR [28] and Samsung Oculus Gear VR HMD’s [27]. Oculus Rift is a commercially available VR HMD with two hand-operated controllers which can be used with commercial computers with appropriate processing and graphics capabilities [10, 28]. The HMD detects head movement and the controllers track hand movements via 3D inertial sensor technology [10]. Case and pilot studies have demonstrated the feasibility of Oculus Rift for research and treatment of acute and chronic pain and movement-based disorders [41–44]. The Samsung Oculus Gear VR HMD [27] is another commercially available unit designed for use with the Samsung Galaxy Series smartphones (i.e., series S6 and newer). The HMD projects VR images are generated by the smartphone with sound [27]. The Samsung Oculus Gear VR HMD has also been utilized in previous research for acute pain management among hospitalized patients [16]. Figure 1 presents research team members demonstrating the Oculus Rift and Samsung Oculus Gear VR (used with written permission).

### Procedures

At CPRP admission, licensed clinical psychologists will inform prospective participants about the study and provide them with a flyer. The clinical staff will then send a VA-encrypted e-mail containing the contact information of any interested Veterans to the study coordinator. A member of the research team (i.e., licensed physical therapist, licensed occupational therapist, or clinical psychology post-doctoral fellow) will follow up with interested Veterans in person to further discuss the study and obtain informed consent. Veterans will be explicitly informed that they can leave the study at any time without penalty and that they can participate in VR as part of their PT even if they choose not to consent for participation in the study.

The CPRP provides two daily group sessions of PT, each session containing up to six Veterans. Within each PT session, participants will be randomized into groups of three. One group will receive the VR intervention for the first 20 min with the other group receiving PT. During VR sessions, one participant will use the Oculus Rift and two participants will use Samsung Oculus Gear VR. During the first VR session, the intensity hierarchy will be explained, and Veterans will begin by using guided meditation (low intensity). In each session, the Veterans will be asked whether they wish to progress to a higher intensity VR APP or continue using similar intensity APPs and their preference to sit or stand during their VR participation. The Veteran will choose whether to sit or stand during their VR participation. After the 20-min VR sessions, the groups will switch treatment modalities (VR to PT and vice versa). Following each VR session, Veterans will be administered the daily rating form. Fear of movement and pain outcomes will be treatment progress measures administered to all Veterans by CPRP staff at admission and discharge. These research teams will retrieve these measures along with demographic information from the VA electronic medical record. Engagement between participants and the research team during VR sessions will help ensure adherence to the intervention protocol. A customizable user manual for Oculus Rift and Samsung Oculus Gear VR was developed for this study to assist in training the research team and to use with the study protocol (available upon request). The study schedule is presented in Table 2.

**Table 2:** Study schedule

CPRP timeline	Admission	Week 1					Week 2					Week 3					Discharge
VR session		1	2	3	4	5	6	7	8	9	10	11	12	13	14	15	
Orientation	X																
Informed consent	X																
Daily rating form																	
APP intensity		X	X	X	X	X	X	X	X	X	X	X	X	X	X	X	
Cybersickness		X	X	X	X	X	X	X	X	X	X	X	X	X	X	X	
Immersion		X	X	X	X	X	X	X	X	X	X	X	X	X	X	X	
Open-ended questions		X	X	X	X	X	X	X	X	X	X	X	X	X	X	X	
Primary measures																	
General feared movements (POQ-VA)	X																X
Common feared movements (FDAQ)	X																X
Secondary measures																	
Pain intensity (POQ-VA)	X																X
Interference with mobility (POQ-VA)	X																X
Interference with ADLs (POQ-VA)	X																X
Negative affect (POQ-VA)	X																X
Pain catastrophizing (PCS)	X																X
Patient-specific functioning (PSFS)	X																X

#### Ethical compliance

The annual internal audit completed by the research compliance office at the James A. Haley Veterans Hospital will serve the purpose of examining ethical compliance. Any adverse events will be reported to clinical staff, the research service at the James A. Haley Veterans Hospital, and the University of South Florida IRB.

#### Confidentiality

Several steps will be taken to secure participant confidentiality. First, any contact information from interested Veterans will be sent from CPRP staff to the research team via VA-encrypted e-mail. Second, electronic data files will be saved only on a secured VA server behind the VA firewall. Third, informed consents will be stored in locked filing cabinets in the project manager’s office. Finally, all raw data will be stored separately in a locked filing cabinet in the principal investigators’ office.

### Primary measures

The daily rating form is available from the authors upon request. Functional and pain measures were chosen based on their psychometric properties, relevance to chronic pain populations, and availability within the CPRP medical record.

#### Feasibility of VR

##### Daily rating form

Following each VR session, a daily rating form will be used to document participants’ VR experiences. Specifically, the research team will use the daily rating form to track the APP the Veteran selects during their VR session, self-reported APP intensity, whether they participate in a seated or standing position, and the number of sessions completed. Information on APP intensity will be used to plot progression across the distraction-to-exposure hierarchy (Aim 1). Veterans will also be assessed using validated single-item measures of “cybersickness” [45] and their level of “immersion” in the VR experience [46]. Information collected from the daily rating form will also be utilized to gain a better understanding of feasibility including potential barriers and facilitators of using VR within this complex Veteran population (Aim 3). For example, chronic pain populations may require special considerations for using VR due to factors such susceptibility to cybersickness (e.g., dizziness, nausea) and physical levels of (dis) comfort of HMDs [47]. Both factors can provoke greater anxiety/panic and be addressed by a tailored approach to HMD selection and content exposure (e.g., lower-intensity stimuli, appropriate VR session length) [47]. Participants will be given the opportunity to provide such additional information about their VR experience via their length of use and four open-ended questions, i.e., likes, dislikes, symptoms, and additional comments. This data will be recorded using the daily rating form and be used to guide any necessary modifications for the main trial (see the “Discussion” section).

#### Fear of movement

Kinesiophobia will be the primary outcome and assessed with the following measures. The first is the two-item “fear” subscale from the Pain Outcomes Questionnaire-VA (POQ-VA) [48], which will be used to examine the general fear of movement (i.e., fear of re-injury, avoidance). The second measure of kinesiophobia is the 10-item Fear of Daily Activities Questionnaire (FDAQ) [49], which will be used to examine common feared movements.

##### Pain Outcomes Questionnaire-VA (primary)

The POQ-VA [48] a comprehensive multidimensional instrument was developed and validated specifically for Veteran populations. The Intake and Discharge versions of this questionnaire contain the following: 19 “primary items” that measure pain treatment outcomes across six prominent pain-related domains including a two-item fear of movement scale. These primary scale items are measured on 11-point rating scales (0 to 10) with higher scores indicating better outcomes. Similar to other scales on this measure, the fear of movement scale has demonstrated good generalizability and discriminant and concurrent validity [48, 50]. Internal consistency may be questionable [48] and no minimum clinically important change (MCIC) standards have been established for this subscale.

##### Fear of Daily Activities Questionnaire

The FDAQ is a self-report measure designed to assess feared *common* activities for people with chronic pain using the Fear-Avoidance Model [49]. All 10-items are measured using a 100-point numeric rating scale ranging from 0 (*no fear*) to 100 (*maximal fear*). The FDAQ can be averaged and utilized as a full scale with item content reflecting upright and seated posture as well as spinal movement. The full-scale FDAQ has demonstrated good internal (Cronbach’s α = .91) and test-retest reliability (ICC = 0.90) as well as strong concurrent validity with disability (*r* = 0.70) and moderate concurrent validity with other pain measures to be used in this study, e.g., Pain Catastrophizing Scale (*r* = 0.52) and the numeric rating scale (*r* = .34) [49, 51]; sensitivity to change with reductions in disability (*r* = 0.49) and pain catastrophizing (*r* = 0.35) at 4-week follow-up following graded-exposure physical therapy [49]. The minimum clinically important change (MCIC) on the FDAQ is a 12.9 point reduction [49].

### Secondary outcome measures

Secondary pain and functional outcomes will be examined to formalize optimal additional measures for VR use in a future RCT.

#### Pain Outcomes Questionnaire-VA (secondary)

The additional 17 primary items on the POQ-VA measure four additional subscales to be examined as *secondary* outcomes. These scales include pain intensity, interference with mobility, interference with ADLs, and negative affect (e.g., depression, anxiety). These subscale scores have demonstrated acceptable internal consistency (Cronbach’s α = 0.78–0.90) [48]. The POQ-VA will also be utilized to collect additional descriptive information including pain history (e.g., chronicity, locations), disability and employment status, opioid use, and pain-related medical utilization over the previous 3 months. No MCIC scores have been published for the POQ-VA subscales except the pain intensity numeric rating scale (MCIC = 2.1–2.8 points) [52]. Daily pain intensity scores will also be collected from the medical record because this may impact progression in the hierarchy.

#### Pain Catastrophizing Scale

The 13-item Pain Catastrophizing Scale (PCS) [53] will be used to measure maladaptive and exaggerated negative beliefs “toward actual or anticipated experiences” of pain (p. 602) [54]. Items are measured on a 5-point Likert-type scale anchored by 0 (*not at all*) and 4 (*all the time*) with higher scores indicating greater levels of catastrophizing. The PCS has demonstrated utility as both a full-scale score as well as a three-factor structure with subscales measuring cognitive “rumination” on pain symptoms (“I keep thinking about how much it hurts”), “magnification” of pain symptoms (“I become afraid that the pain will get worse”), and “helplessness” (“There is nothing I can do to reduce the intensity of the pain”) [53, 55]. The full-scale PCS score has established MCIC improvements for return to work (38%) and pain reduction (44%) following rehabilitation [56], but not for its subscales. Hence, the full-scale PCS score will be examined in the current study. The full PCS has demonstrated acceptable internal consistency (Cronbach’s α = 0.87–0.93) [53, 55], test-retest reliability (ICC = 0.75) [53], and criterion validity in differentiating between chronic pain outpatient and community adult samples [55].

#### Patient-Specific Functional Scale

The Patient-Specific Function Scale (PSFS) [57] can be tailored to the individual’s health-related functioning. Participants are asked to self-select three-to-five activities that cause great difficulty or they can no longer engage in secondary to a specific health condition. They are then asked to rate the difficulty of these activities on an 11-point scale anchored by 0 (*unable to perform*) and 10 (*able to perform at prior level*). A score will be obtained by averaging activities and using reverse-scoring for interpretation consistency with other measures; higher scores indicate worse outcomes. The PSFS has demonstrated good test-test reliability (ICC = 0.82) and sensitivity to change among people with chronic neck pain [58] and acute low back pain (ICC = 0.91–0.97) [59], convergent validity with disability (*r* = 0.55–.74), role functioning (*r* = 0.44), physical functioning (*r* = 0.30), and bodily pain (*r* = 0.34) [59]. The MCIC for the PSFS has been established for small (1.3–2.29 points), moderate (2.3–2.69), and large clinical improvements (> 2.70) [60].

### Analytic plan

All demographic characteristics, primary and secondary measures, will be described by the use of means and standard deviations for continuous variables and percentages for categorical variables. Multiple steps will be taken to handle missing data. Missing value patterns will be examined using Little’s Missing Completely at Random Test [61]. Missing data will be estimated using multiple imputations with demographic information and participant baseline scores on primary and secondary measures as predictors of missing items [62]. Up to 20% of missing data will be allowed for the proposed study based on evidence suggesting that standardized bias of mean changes is acceptable when using multiple imputation techniques in a small sample (*N* = 20) [63].

#### Aim 1

Describe the individual Veteran trajectories and APP intensity ratings across the proposed distraction-to-exposure hierarchy. Distributions of the Veteran-selected VR APPs (proposed movement intensity range of 1–6) will be calculated and plotted across all sessions over the course of the study. The frequency of individual Veteran trajectories will be counted to identify common patterns for the preferred level of movement. Veteran’s average self-reported intensity ratings for individual VR APPs will be plotted across sessions. Consistencies between the level of movement required to engage in the APP and self-reported intensity will be descriptively examined.

#### Aim 2

Estimate the within-subjects effect size and 95% CI for changes in fear of movement to provide insight into the likely magnitude of effect associated with the VR intervention. For the primary outcome (POQ-VA: fear of movement) the within-participant effect size calculations (*M*_post_ – *M*_pre_/SD_diff_) will be made along with 95% CIs to provide insight into the effect associated with VR utilization [64]. This step will also be completed for the FDAQ. Furthermore, the FDAQ mean difference from baseline and 95% CI will be calculated and compared to the established MCIC for this measure. The proportion of Veterans that experience clinically meaningful change will then be calculated. For secondary pain and functional measures, the effect sizes, mean differences from baseline with 95% CIs, and the proportion of participants that experience meaningful change will be examined. Data from secondary measures will be used to select optimal instruments for use with VR in a larger RCT.

#### Aim 3

Pilot test this protocol to assess the feasibility of VR use to plan for a future RCT. Veteran experiences using VR as an adjunct for pain including potential barriers and facilitators to use will be examined. Feedback and responses to questions on the daily rating form will be transcribed word-by-word and analyzed using the following steps [65]. First, the text will be read several times. Second, exploratory commenting will be performed line-by-line to let the data drive the coding. Third, line-by-line coding will be chronologically ordered into emergent themes. Steps one through three will be tabled: a MS Word document will be created for each emergent theme identified in steps one through three. Super-ordinate themes will be identified by searching for patterns and connections between the emerging themes. Compliance to the VR protocol will also be examined. The proportion of VR sessions attended (attended sessions/total sessions) will be calculated. Additionally, the number of VR sessions completed without early termination (completed sessions/total session attempts) will be calculated and reported. This information will help us determine anticipated adherence for the larger trial.

## Discussion

Studies have demonstrated the efficacy of using VR interventions as a means of pain distraction for people with acute [11, 16, 42] and to a lesser extent chronic pain [17]. Similarly, evidence suggests that graded exposure to feared movements via VR interventions can improve function among people with chronic pain [21, 22]. However, there is a lack of sufficient evidence from studies examining multiple VR APPS or varying simulation intensity levels, both of which have been identified as important directions for future VR research [16]. Moreover, no studies have examined distraction and exposure methods as part of a two-dimensional VR hierarchy. The proposed research addresses these gaps in the literature by utilizing multiple VR APPs requiring varying levels of movement that range from low-intensity pain distraction to a more active exposure to movement.

This study will inform the feasibility of a larger RCT examining the clinical utility of using VR to reduce fear of movement, pain, and increase function among Veterans with chronic pain. This will be accomplished in multiple ways. First, effect size estimates for improvement in fear of movement will be used to power a larger RCT. Second, effect size and clinically meaningful change on secondary outcomes will help in the selection of optimal scales for use with VR in the larger clinical trial [66]. Third, descriptive information collected on Veteran trajectories for the proposed hierarchy (i.e., APP selection, self-reported APP intensity) will assist in any necessary modifications so that Veterans are not over- or underexposed. Fourth, data collected using the daily rating form will provide useful information regarding Veterans with chronic pain using this technology. This will include whether 20 min is an appropriate length for VR exposure, experienced adverse events (e.g., cybersickness, falls), and facilitators (e.g., immersion, HMD preferences) or barriers (e.g., physical, psychological discomfort) to VR use [47, 66]. Additional qualitative information will help with the identification of any additional unforeseen factors.

Examining the feasibility of this protocol will also be beneficial for Veterans, clinicians, and policymakers. Per the 2016 National Pain Strategy [67], the federal government’s first coordinated plan strives to reduce the burden of chronic pain in the USA. Variations in clinical practice and inadequate tailoring of pain therapies, as well as a reliance on relatively ineffective high-risk treatments, have contributed to the poor quality of care for people with pain [67]. If the aims of this research are achieved, VR will be used in combination with established pain management strategies to decrease pain and opioid use. VR has the advantage of being easily implemented both within VA healthcare settings as well as in Veterans’ own residences, where engagement in ongoing self-management approaches is often most challenging.

VR therapies are projected to have a $3.9 billion market size by 2023 [68], yet despite this tremendous public health burden, published research to date has not extended beyond pilot trials and case studies. Given the lack of large-scale RCTs examining the clinical effectiveness of VR, findings from the proposed study will present a key step to inform a larger RCT to validate our proposed hierarchy and compare it to an active control group (i.e., VR APPs with no known therapeutic value). This level of RCT evidence represents a necessary next step in the evolution of clinical VR research.

## Data Availability

Once completed, the final de-identified datasets from this study (qualitative and quantitative) and the VR user manual will be made available by the corresponding author upon reasonable request.

## Abbreviations

ADL: Activities of daily living
CBT: Cognitive behavioral therapy
CPRP: Chronic Pain Rehabilitation Program
FDAQ: Fear of Daily Activities Questionnaire
HMD: Head-mounted display
PCS: Pain Catastrophizing Scale
POQ-VA: Pain Outcomes Questionnaire-VA
PSFS: Patient-Specific Functional Scale
PT: Physical therapy
RCT: Randomized controlled trial
VA: Veterans Affairs
VR: Virtual reality
